# Assessing patients’ characteristics and treatment patterns among children with atopic dermatitis

**DOI:** 10.1186/s13052-021-00987-9

**Published:** 2021-04-16

**Authors:** Davide Geat, Mattia Giovannini, Gabriele Barlocco, Riccardo Pertile, Manuela Pace, Francesca Mori, Elio Novembre, Giampiero Girolomoni, Mario Cristofolini, Ermanno Baldo

**Affiliations:** 1 Department of Medicine, Section of Dermatology and Venerology, University of Verona, Verona, Italy; 2 Allergy Unit, Department of Pediatrics, Meyer Children’s University Hospital, Florence, Italy; 3 “Giovan Battista Mattei” Research Institute, Stenico, Italy; 4 Department of Clinical and Evaluative Epidemiology, Trento Health Service, Trento, Italy; 5 Department of Pediatrics, S. Maria del Carmine Hospital, Rovereto, Italy

**Keywords:** Atopic dermatitis, Comano thermal spring water, Emollients, Food allergy, Children

## Abstract

**Background:**

Atopic dermatitis (AD) is the most common immune-mediated skin disease in childhood. Several treatment options for pediatric AD, both topical and systemic, are currently available. We carried out a single-center observational study with the aim of describing characteristics and treatment patterns in pediatric AD patients.

**Methods:**

The study included 867 patients aged ≤16 years (females 50.5%, mean patient’s age 5.9 years, standard deviation ±3.6 years) with a previous doctor-confirmed diagnosis of AD who underwent balneotherapy at the Comano Thermal Spring Water Center (Comano, Trentino, Italy) from April to October 2014.

**Results:**

Among the patients included in the study, 41.2% had mild (SCORing Atopic Dermatitis, SCORAD 0-15), 43.6% moderate (SCORAD 16–40) and 15.2% severe AD (SCORAD > 40). A higher occurrence of reported food allergy was observed among children with more severe AD **(***p* < 0.0001), while no association was found between AD severity and reported inhalant allergy or passive smoking (*p* = 0.15 and 0.92, respectively). Emollients (55.1%) and topical corticosteroids (TCS; 45.7%) were the main treatment options used in the previous month. The use of oral steroids and topical calcineurin inhibitors (TCI) was considerably less common (6.3 and 4.5%, respectively), while no patients were on systemic agents other than steroids. Among patients with severe AD, 9.8% had not used TCS, TCI or any systemic treatments. Moreover, 20.0% of the patients in the study population had followed elimination diets, although only 27.2% of them had a reported food allergy.

**Conclusions:**

A significant difference in the prevalence of reported food allergy emerged across the different AD severity categories. Furthermore, although further data are necessary to confirm our findings, undertreatment in children with AD appeared to be very common, at least among those attending the Comano Thermal Spring Water Center. Moreover, many patients followed elimination diets in the absence of reported food allergy.

## Introduction

Atopic dermatitis (AD) is the most common chronic immune-mediate skin disease in childhood, with a lifetime prevalence ranging between 15 and 20% [1]. Because of its clinical manifestations and its association with psychological stress, sleeping disturbances and poor performance at school, the impact of AD on children’s quality of life is considerable [2]. Topical treatments are the main management options for mild to severe AD in children. They include emollients, topical corticosteroids (TCS), topical calcineurin inhibitors (TCI) and, currently, crisaborole, which is mostly used in the United States. Emollients play a crucial role, as they contribute to restore the skin barrier, whose anomalies are a driving factor for AD. In fact, decreased function of the skin barrier due to genetic (e.g., filaggrin deficiency) or acquired factors can lead both to increased allergen penetration and easier skin irritation, which contribute to skin inflammation in AD. On the other hand, TCS exert an anti-inflammatory function that is crucial to control the acute flares of the disease [3]. Moreover, an intermittent use of TCS on skin sites where AD tends to recur (i.e., proactive therapy) can reduce the number of relapses [4]. However, despite their efficacy, adherence to TCS is often suboptimal because of the anxiety and fears of patients and their caregivers [5, 6]. “Corticosteroid phobia” was the subject of a recent systematic review that included 16 studies and reported a prevalence ranging between 21.0 and 83.7% worldwide [7]. This phenomenon is rooted in patients’ irrational fears of TCS-triggered skin side effects [8, 9]. Finally, severe AD in children and adolescents can also be treated with several systemic options including steroids, cyclosporin and dupilumab [3, 10].

## Methods

We carried out a single-center observational study with the aim of describing patients’ characteristics and treatment patterns among Italian pediatric patients with AD. The study was conducted at the Comano Thermal Spring Water Center (Comano, Trentino, Italy) on patients aged ≤16 years who underwent balneotherapy from April to October 2014. Patients with previous doctor-confirmed diagnosis of AD using the criteria of Hanifin and Rajka [11] were included in this study. As required by the principles of good clinical practice in thermal medicine and by the institute’s policy [12], we considered any coexisting systemic pathology (e.g., infections, heart diseases, immunodeficiencies or malignancies) or cutaneous disease (e.g., viral, bacterial or fungal infections, skin cancer or ulcers) as exclusion criteria for the study. In fact, such pathologies make balneotherapy treatment inadvisable, as well. An informed, written consent was obtained from all parents/guardians before including the patients in the study.

During the admission visits to the Comano Thermal Spring Water Center, demographic and clinical data were collected through a physical exam and a doctor interview: AD severity, age of onset, history of AD in first-grade relatives, exposure to passive smoking, coexisting reported inhalant or food allergies. Treatments, including topical and systemic, used by patients in the previous month were recorded as well. AD severity was recorded during the admission visit using the following five SCORAD (SCORing Atopic Dermatitis) categories: 0–15, 16–30, 31–40, 41–60, > 60. SCORAD includes both objective items – percentage of the affected skin surface area (A; 0–100), intensity (B; 0–3 points are assigned to each of the following elements: dryness, erythema, oozing/crusting, edema/papulation, excoriation, lichenification) – and subjective items (C; 0–10 points for each of the following elements: pruritus and sleeplessness). Individual item scores are then combined according to the following formula: A/5 + 7B/2 + C. The minimum and maximum SCORAD are therefore 0 and 103, respectively [13].

To describe the results from a statistical point of view, qualitative data were presented as frequency tables (number of observations or percentages) and/or through histograms, while quantitative data were expressed as number of observations, mean and standard deviation (SD). Chi-squared test with Yates correction was used to evaluate associations between AD severity (expressed through SCORAD categories) and qualitative variables. The differences were considered statistically significant with *p* ≤ 0.05. Statistical data analysis was carried out using SAS software (SAS institute, Cary, North Carolina, United States of America).

## Results

We recruited 867 patients whose mean age was 5.9 ± 3.6 years; 50.5% of them were females (Table 1).

**Table 1:** Characteristics of the study population

Characteristics (***n*** = 867)	
Age (years)	5.9 ± 3.6
Females	438 (50.5%)
SCORAD:	
0–15	357 (41.2%)
16–30	237 (27.3%)
31–40	141 (16.3%)
41–60	91 (10.5%)
> 60	41 (4.7%)
Age of onset	
< 2 months	229 (26.4%)
2–6 months	242 (27.9%)
7–12 months	154 (17.8%)
13–36 months	149 (17.2%)
> 36 months	93 (10.7%)
History of AD in first-grade relatives	35 (4.0%)
Exposure to passive smoking	134 (15.5%)
Reported inhalant allergy	199 (23.0%)
Reported food allergy	82 (9.5%)
Therapies in the last month:	
Emollients	478 (55.1%)
TCS	396 (45.7%)
1–5 days/month	120 (13.8%)
6–10 days/month	116 (13.4%)
> 10 days/month	160 (18.5%)
TCI	39 (4.5%)
Oral steroids	55 (6.3%)

Data were represented as N (%) or mean ± standard deviation

*AD* atopic dermatitis, *SCORAD* SCOring Atopic Dermatitis, *TCI* topical calcineurin inhibitors *TCS* topical corticosteroids

The percentages of patients with SCORAD 0–15, 16–30, 31–40, 41–60 and > 60 were 41.2, 27.3, 16.3, 10.5 and 4.7% respectively. Thus, 41.2% of the patients had mild AD (SCORAD 0-15), 43.6% had moderate AD (SCORAD 16–40) and 15.2% had severe AD (SCORAD > 40).

In 54.3% of the children, AD had first appeared before 6 months of age. Specifically, in 26.4% of them, the onset of AD had been reported before the age of 2 months, while in 27.9% it had been reported between the age of 2 and 6 months. 17.8% of the children were first affected by AD at the age of 7–12 months. A similar percentage of children (17.2%) had developed AD at the age of 13 months-3 years, while only 10.7% had developed AD after the age of 3 years.

A history of AD in first-grade relatives was reported in only 4.0% of the group, while coexisting reported inhalant or food allergies were recorded in 23.0% (mean age 7.9; SD ± 2.6 years) and 9.5% (mean age 5.8; SD ± 3.6 years), respectively. Exposure to passive smoking was recorded in 15.5% of the patients.

**Fig. 1:**
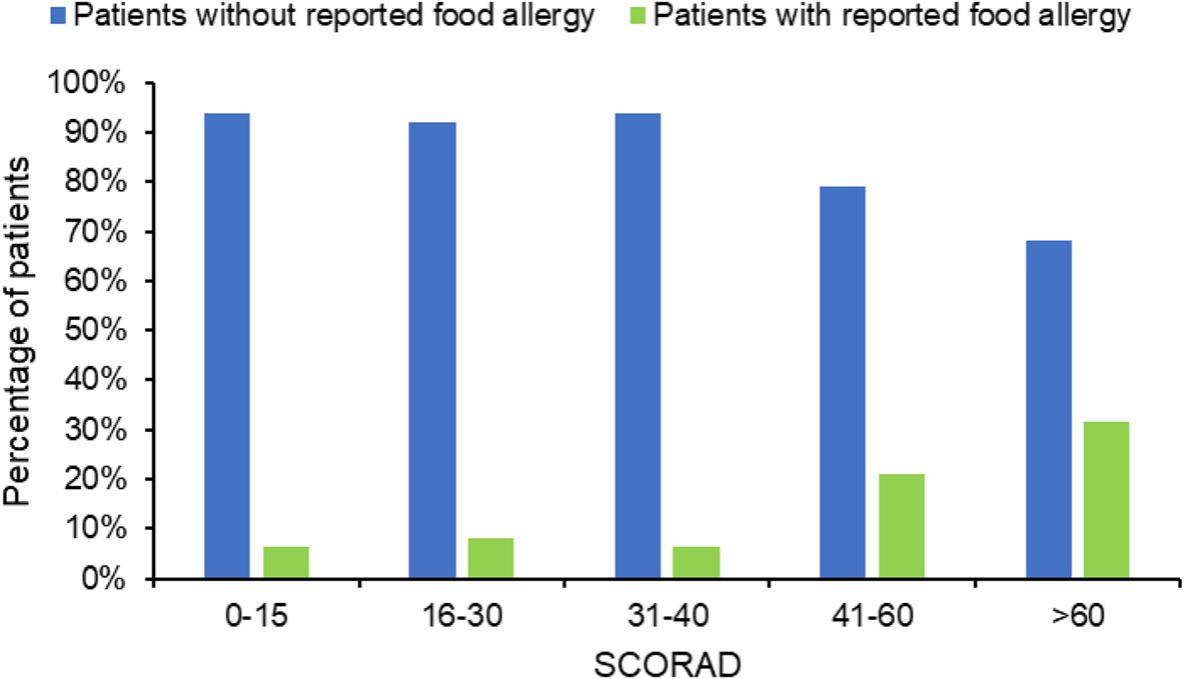
Prevalence of reported food allergy according to the severity of atopic dermatitis. SCORAD SCORing Atopic Dermatitis

A difference in the occurrence of reported food allergy was observed across the different AD severity categories (*p* < 0.0001; Fig. 1), ranging from 6.2% among patients with SCORAD 0–15 to 31.7% in those with SCORAD > 60. No differences about the occurrence of passive smoking exposure and reported inhalant allergy were observed across the severity categories (*p* = 0.92 and 0.15, respectively; data not shown).

**Fig. 2:**
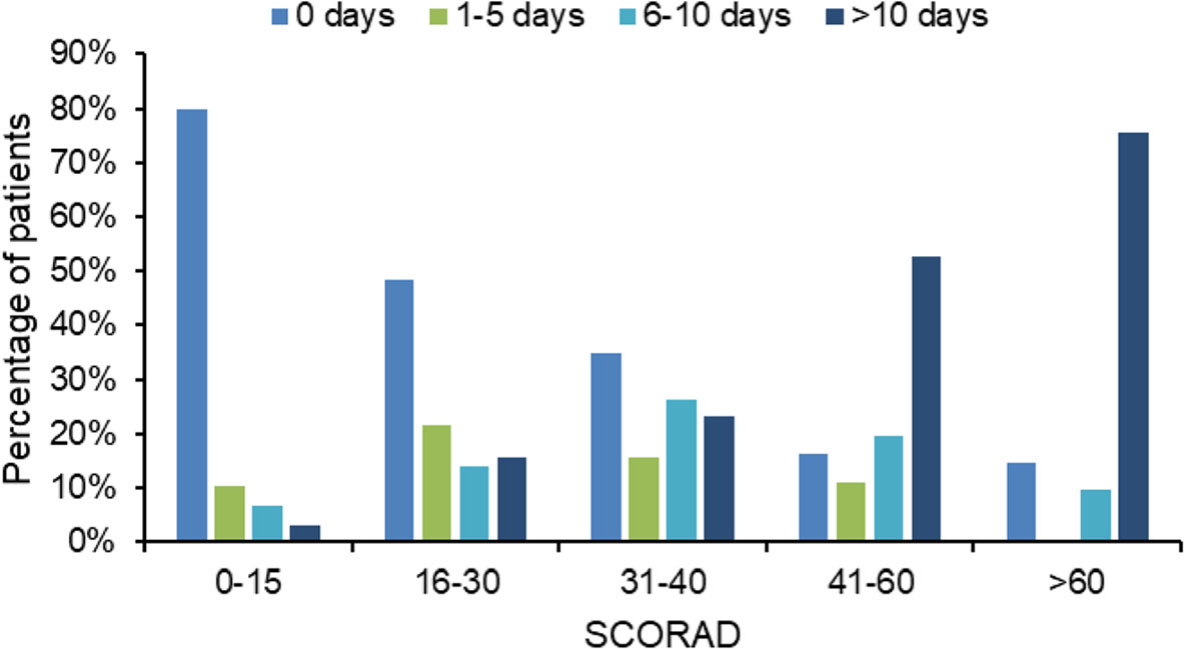
Days of topical corticosteroids in the previous month according to the severity of atopic dermatitis. SCORAD SCORing Atopic Dermatitis

The AD treatments used in the previous month were, in order of frequency, as follows: emollients (55.1%), TCS (45.7%), oral steroids (6.3%) and TCI (4.5%). No systemic therapies other than oral corticosteroids were reported. The percentage of emollient users was slightly higher in patients with severe disease than in those with mild disease: 61.4% in those with severe AD vs 59.0% (moderate AD) and 48.7% (mild AD) (*p* < 0.01, data not shown). No association between the use of emollients and TCS was observed (data not shown). Based on our data, 13.8% of the study population had applied TCS less than 5 days in the previous month and a similar percentage (13.4%) in 6–10 days, whereas 18.5% had applied TCS more than 10 days (Table 1). Higher disease severity was associated with higher TCS use (*p* < 0.0001). For example, only 3.1% of patients with SCORAD 0–15 had applied TCS more than 10 days in the previous month, the corresponding percentage in patients with SCORAD > 60 being 75.6% (Fig. 2). Conversely, 79.8 and 10.4% of those with mild disease had used respectively no TCS or a 1–5 days TCS treatment in the preceding month, compared to 14.6 and 0.0% in patients with SCORAD > 60. Among patients with severe AD, 36.6% had been treated with systemic steroids in the previous month, 85.4% with TCS and 12.2% with TCI, while 9.8% had not used systemic steroids, TCS or TCI at all.

Moreover, 20.0% of the study population had followed elimination diets, although only 27.2% of those who were on elimination diets had a reported food allergy. On the contrary, 80.0% of the study population had not followed any elimination diets, although 5.0% of those who were not on elimination diets had a reported food allergy (Table 2).

**Table 2:** Use of elimination diets according to reported food allergy

Elimination diets	Patients without reported food allergy	Patients with reported food allergy	Total
No	659 (95.0%)	35 (5.0%)	694 (80.0%)
Yes	126 (72.8%)	47 (27.2%)	173 (20.0%)
Total	785 (90.5%)	82 (9.5%)	867 (100.0%)

Data were represented as N (%)

## Discussion

In our study, a significant difference in the occurrence of reported food allergy emerged across the different AD severity categories. This seems to confirm previous studies in which severe vs mild to moderate AD in children was associated with a higher one-year prevalence of food allergy (27.0% vs 14.1%) [14]. Skin barrier defects play a pivotal role in the pathogenesis of AD, and our findings mirror previously-published studies suggesting that skin barrier impairment, coupled with cutaneous allergen exposure, may be crucial in the development of food allergy and allergen sensitization through the skin (i.e., “dual-allergen exposure hypothesis”) [15, 16]. Little evidence is currently available on the association between AD severity and passive smoking in children. In a study on 100 Greek children, parental passive smoking was associated with severe AD (aOR: 4.6; 95% CI: 1.0–22.1; *p* = 0.050). However, a weaker association with severe AD was found, if compared to the other risk factors evaluated (excessive cleanliness *p* < 0.001, aOR: 59.4; 95% CI: 10.9–322.6; Radioallergosorbent Test, RAST > 0.7 kU/l *p* = 0.014, aOR: 7.9; 95% CI: 1.5–41.0) [17]. In our study, no differences in the occurrence of passive smoking exposure and reported inhalant allergy were found across the AD severity categories (*p* = 0.92 and 0.15, respectively).

As for the treatment patterns, 9.8% of patients with severe AD had not been treated with TCS, TCI or any systemic steroids in the previous month. Such a figure underlines the magnitude of undertreatment among patients with severe AD, at least in those attending the Comano Thermal Spring Water Center. In a country like Italy, where the National Health System guarantees free unlimited access to pediatric primary care, AD undertreatment might largely be explained by limited access to medical care due to personal beliefs (e.g., seeking alternative treatments) or by low adherence (due to “corticophobia”, forgetfulness or other reasons). Additionally, 36.6% of patients with severe AD had been treated with systemic steroids in the previous month. Interestingly, although short courses of systemic steroids are an effective and inexpensive treatment, the latest European and Italian guidelines on the management of AD recommend a limited use because of their unfavorable risk-benefit ratio. Moreover, it is stated there that the indication for oral steroids in children should be handled even more cautiously than in adults [3, 10, 18–20]. It is also worth noting the limited use of emollients in our study population: only 55.1% of patients resorted to them in the previous month, their occurrence being slightly higher in patients with severe disease. Few studies have reported the prevalence in the use of emollients in pediatric patients with AD. A Polish study [21] has showed a prevalence of emollients use of 82% in AD patients. Still, the small study population (22 patients, adults and children) makes it difficult to generalize these findings. It is important to point out that emollients are an essential element in the treatment of AD. In fact, in several studies, regular use of emollients has proved to achieve a short-term steroid sparing effect in mild to moderate AD [22] and long-term use of emollients is recommended for the maintenance of stable disease in the latest European and Italian guidelines [3, 18–20]. Health economic analyses showed that the use of emollients is cost-effective compared with a no-treatment strategy [23]. Several factors may explain our findings. First, as the Italian National Health System does not cover emollients, their cost could represent a hindrance to their use. Secondly, parents’ poor knowledge on the long-term efficacy of emollients in preventing AD flare-ups may also lead to underuse. Indeed, a recent interview study [24] showed that parents had mixed views on long-term emollient use to prevent exacerbations. The authors concluded that providing a rationale for long-term emollient use could help improve adherence. Educational programs and adequate time to allow for patient education during routine visits may also help raise awareness about the utility of emollients in managing AD.

Finally, it is interesting to point out in our study how surprisingly only 27.2% of patients on elimination diets had a reported food allergy. It is also interesting to highlight how 5.0% of patients who were not on elimination diets in our study had a reported food allergy. While elimination diets are indeed critical in AD patients with food allergy, it seems fundamental for pediatricians and dermatologists to promote an evidence-based management of suspected adverse reactions to food [25–27] based on a rigorous diagnostic allergy work-up carried out according to the current guidelines [28]. Indeed, an incorrect management of food allergy can lead to inappropriate and dangerous dietary restrictions - especially in children, who are very vulnerable to nutritional deficiencies [29] or to the risk of reactions [30–32].

The main limitations of our study are mostly due to the clinical setting in which it was carried out, i.e., admission visits for balneotherapy. Indeed, the characteristics and treatment patterns in pediatric AD cases recorded at the Comano Thermal Spring Water Center may not be entirely representative of the general Italian pediatric population. Moreover, food and inhalant allergy was reported, but no rigorous diagnostic allergy work-up (based on the use of prick test, specific IgE or food challenge) nor other laboratory investigations were performed at the Comano Thermal Spring Water Center, which is not the appropriate clinical setting to carry them out. Lastly, data on antihistamine use were not collected. However, the large population size is undoubtedly the strongest point of our study, as it enabled us to describe the characteristics and treatment patterns in pediatric AD cases.

## Conclusions

A significant difference in the prevalence of reported food allergy emerged across the different AD severity categories. Furthermore, although further data remain necessary to confirm our preliminary findings, undertreatment in children with AD was observed - at least in patients attending the Comano Thermal Spring Water Center. Moreover, many patients followed elimination diets in the absence of reported food allergy.

## Data Availability

The access to datasets generated and/or analyzed during the current study is not publicly available due to the Center’s research policy to guarantee the privacy of the participants, whose research data are confidential. Aggregate analyses are however available on reasonable request to the corresponding author.

## Abbreviations

AD: Atopic Dermatitis
CI: Confidence Interval
OR: Odds Ratio
RAST: Radioallergosorbent Test
SCORAD: SCORing Atopic Dermatitis
SD: Standard Deviation
TCS: Topical Corticosteroids
TCI: Topical Calcineurin Inhibitors
